# 10-Year Molecular Surveillance of *Listeria monocytogenes* Using Whole-Genome Sequencing in Shanghai, China, 2009–2019

**DOI:** 10.3389/fmicb.2020.551020

**Published:** 2020-12-15

**Authors:** Hongzhi Zhang, Weijie Chen, Jing Wang, Biyao Xu, Hong Liu, Qingli Dong, Xi Zhang

**Affiliations:** ^1^ Shanghai Municipal Center for Disease Control and Prevention, Shanghai, China; ^2^ Minhang District Center for Disease Control and Prevention, Shanghai, China; ^3^ School of Medical Instrument and Food Engineering, University of Shanghai for Science and Technology, Shanghai, China

**Keywords:** *Listeria monocytogenes*, whole-genome sequencing, serogroup, multilocus sequencing typing (MLST), cgMLST

## Abstract

*Listeria monocytogenes* is an etiologic agent of listeriosis, and has emerged as an important foodborne pathogen worldwide. In this study, the molecular characteristics of 155 *L. monocytogenes* isolates from seven food groups in Shanghai, the biggest city in China, were identified using whole-genome sequencing (WGS). Most *L. monocytogenes* isolates (79.3%) were obtained between May and October from 2009 to 2019. The serogroups and clonal complexes (CCs) of *L. monocytogenes* were found useful for identifying potential health risks linked to foods. Differences in distributions of serogroups and CCs among different food groups were analyzed using *t*-test. The results showed that the IIa and IVb serogroups were identified among most of food groups. However, the prevalence of serogroup IIb was significantly higher in ready-to-eat (RTE) food and raw seafood than in other food groups, similar to group IIc in raw meat and raw poultry than others. Meanwhile, the prevalence of CC9 in raw meat and raw poultry, CC8 in raw poultry, and CC87 in raw seafood significantly exceeded that of in other food groups. Specially, CC87 was the predominant CC in foodborne and clinical isolates in China, indicating that raw seafood may induce a high-risk to food safety. Also, hypervirulence pathogenicity islands LIPI-3 and LIPI-4 were found in CC3, CC1, and CC87, respectively. The clonal group CC619 carried LIPI-3 and LIPI-4, as previously reported in China. Core genome multilocus sequence typing (cgMLST) analysis suggested that CC87 isolates from the same food groups in different years had no allelic differences, indicating that *L. monocytogenes* could persist over years. These 10-year results in Shanghai underscore the significance of molecular epidemiological surveillance of *L. monocytogenes* in foodborne products in assessing the potential risk of this pathogen, and further address food safety issues in China.

## Introduction

*Listeria monocytogenes* is an etiologic agent of listeriosis, a significant public health problem in many countries, due to its high case fatality rate (Thomas et al., 2013), and is also an important foodborne pathogen in China (Li et al., 2018; Yin et al., 2020). Listeriosis outbreaks have been reported in many countries. Chinese national Listeriosis surveillance data collected between 2013 and 2017 showed that 211 listeriosis cases were diagnosed from 64 sentinel hospitals (Li et al., 2019), of which 64.5% were perinatal cases. The Universal Two-Child Policy was implemented in 2015, and has probably increased the number of pregnancies and births in China. Due to the introduction of this policy, listeriosis incidence might increase accordingly.

Listeriosis cases are linked to various food products, but are predominantly associated with ready-to-eat (RTE) foods with long shelf lives (Andrea et al., 2015). The long and variable incubation period of the illness indicates frequent difficulty in establishing links between cases and suspected food items. The correlation between foods and listeriosis cases in China has not yet been clarified. Li et al. (2019) performed an epidemiologic investigation on 61 listeriosis cases, and found that *L. monocytogenes* isolates from suspected foods were unlinked to clinical isolates, using pulsed-field gel electrophoresis (PFGE) and core genome multilocus sequence typing (cgMLST). Although no reported outbreaks of listeriosis have yet been directly attributed to the consumption of raw meat, poultry, or seafood, these foods can cause cross-contamination of other foods, and *L. monocytogenes* might survive in processed products (Luo et al., 2019; Chen et al., 2020a). Therefore, a high number of *L. monocytogenes* isolates in these foods poses a potential risk, which may induce listeriosis occurrence.

Molecular typing of *L. monocytogenes* isolates from foods and clinical cases is used to establish links between listeriosis cases and food items, and assists in tracing the original source of contamination (Chen et al., 2013). Studies have reported that certain serotypes and clonal complexes (CCs) are commonly encountered in clinical cases (Chenal-Francisque et al., 2011; Maury et al., 2016).

Whole-genome sequencing (WGS) has been used to epidemiologically investigate listeriosis outbreaks (Pietaka et al., 2019). Different WGS-based analytical approaches have been used for *L. monocytogenes* typing, including cgMLST, which produces higher accuracy and discrimination than PFGE and MLST (Moura et al., 2016).

In China, *L. monocytogenes* surveillance in food products was launched in 2000 (Pei et al., 2015). In this study, 155 *L. monocytogenes* isolates from 2009 to 2019 were analyzed. This study verified the molecular characteristics, including cgMLST, serogroup, ST, and LIPI, of *L. monocytogenes* isolates found in seven food groups available in Shanghai, and identified potentially high-risk foods causing listeriosis, and also supplied data for food safety risk assessment by comparing these characteristics with those of foodborne and clinical isolates around the world.

## Materials and Methods

### Bacterial Strains and Growth Conditions

In this study, 155 *L. monocytogenes* isolates from foods were analyzed. According to the Chinese national microbiological food safety surveillance network plan, foods from 22 different categories were collected monthly, and analyzed for the presence of *L. monocytogenes* (Supplementary Table S1) using the Chinese national standard GB4789.30 (2016) method. *Listeria monocytogenes* ATCC19115 as the reference strain was used in the study.

### Whole-Genome Sequencing

Genomic DNA was extracted using DNeasy Blood & Tissue Kits (Qiagen, Germany) according to the manufacturer’s protocol, with minor changes. The cells were prelyzed with lysozyme for 30 min at 37°C, and proteinase K treatment was extended to 30 min. DNA concentration, quality, and integrity were determined using a Qubit Flurometer (Invitrogen, United States) and a NanoDrop Spectrophotometer (Thermo Scientific, United States). Sequencing libraries were generated using the TruSeq DNA Sample Preparation kit (Illumina, United States). Genome sequencing (GS) was performed using the Illumina HiSeq platform (Illumina, United States). The reads were trimmed and assembled using the CLC Genomics Workbench v7.0 (CLC Bio, Aarhus, Denmark).

### Core Genome MLST *in silico* Subtyping

All isolates were typed using cgMLST as implemented in BioNumerics V7.6 software (Applied Maths, Belgium): cgMLST (based on profiles of 1,748 coding loci in the BigsDB Pasteur cgMLST scheme, https://bigsdb.pasteur.fr/listeria/listeria.html). For cgMLST analysis, assembly-based and assembly-free allele calling were performed using the default settings, and dendrograms based on summary calls were constructed by applying categorical similarity coefficients and the unweighted pair group method with arithmetic average (UPFMA).

### Serogroup Determination

Four major serogroups (IIa: serotypes 1/2a, 3a, 3c; IIb: 1/2b, 3b, 7; IIc: 1.2c, 3c; and IVb: 4b, 4d, 4e) could be identified using specific genes: *lmo0737*, *lmo1118*, ORF2819, ORF2110, and *prs* (Burall et al., 2015).

The sequence data for the five genes for *L. monocytogenes* were extracted from the genome data. Serogroups were determined by the presence or absence of the five genes using BLAST.

### MLST Determination

Sequence types (STs) and CCs were assigned using BioNumerics software (version 7.7 Applied Maths, Kortrijk, Belgium) according to the traditional seven housekeeping loci MLST scheme (Ragon et al., 2008), and sequence data of the isolates were extracted from their genome data.

### Virulence Profiles

To identify LIPI-1, LIPI-2, and LIPI-4, the assembled genomes of the isolates in this study were input into the Virulence Factor Database (VFDB; MOH Key Laboratory of Systems Biology of Pathogen, Institute of Pathogen Biology, Beijing, China; http://www.mgc.ac.cn). LIPI-4 comprises of a cluster of six genes (GlvA, Gat-pr, YdjC, GatA, GatB, and GatC). LIPI-4 has recently been identified as a pathogenic island (Maury et al., 2016).

### Statistical Analysis

We analyzed the associations between the four serogroups and different food groups, and the CCs and food groups, by calculating prevalence ratios and *p* value. The analysis was performed using the *t*-test, where *p* < 0.05 was considered statistically significant difference (Chen and Tang, 1994).

## Results

### Food Groups and Serogroups

A total of 155 *L. monocytogenes* strains were analyzed. These strains were isolated from 2009 to 2019 from various food groups, including RTE food (*n* = 31), raw poultry (*n* = 45), raw meat (*n* = 42), Chinese RTE food (*n* = 13), raw seafood (*n* = 20), rice and flour products (*n* = 3), and eggshell (*n* = 1; Supplementary Table S1). The RTE foods mainly comprised sashimi, salads, and cooked meat products (77.4%). Raw poultry, raw meat, and raw seafood comprised fresh, frozen, chilled, and defrosted products. Chinese RTE foods were Chinese style cold dishes with vegetables and meat consumed without heating. Three *L. monocytogenes* isolates were obtained from rice and flour products, and one was obtained from eggshell.

**Figure 1:**
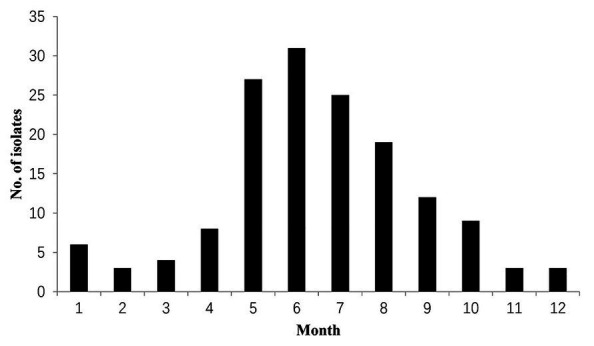
Distribution of *Listeria monocytogenes* in different months. *Listeria monocytogenes* isolates were detected from 2009 to 2019.

These *L. monocytogenes* isolates were obtained from January to December every year (Figure 1). Most *L. monocytogenes* (79.3%) isolates were observed from May to October (2009–2019).

### Correlations Between Food Groups and Serogroups

**Figure 2:**
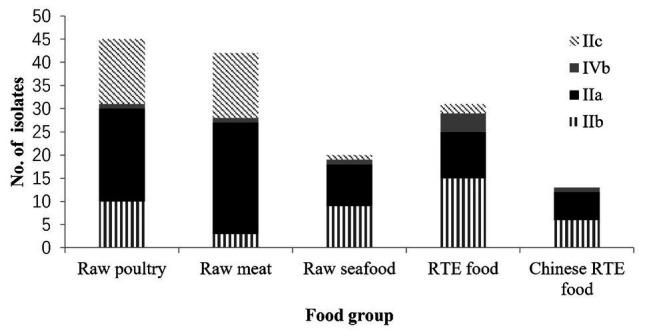
Distribution of four serogroups (IIa, IIb, IIc, and IVb) in seven food groups.

Figure 2 shows the four main serogroups detected in 155 foodborne *L. monocytogenes* isolates. The predominant serogroup was IIa (44.5%, 69/155), followed by IIb and IIc (28.4 and 21.3%, respectively), and nine *L. monocytogenes* isolates were in the IVb serogroup (5.8%). The differences in distributions of serogroups among different food groups were analyzed using the *t*-test (Table 1). The differences in distributions of serogroup IIa among the different food groups were insignificant. However, the distribution of serogroup IIb in RTE food significantly exceeded that of non-RTE food (*p* < 0.05). The distribution of serogroup IIc in raw meat and raw poultry significantly exceeded that of other food groups (*p* < 0.05). No significant difference in the distribution of serogroup IVb for the three foods was observed. Two food groups: rice and flour products, and eggshell could not be analyzed using a *t*-test, because the number of isolates was insignificant for statistical analysis.

**Table 1:** Pairwise association for *L. monocytogenes* serogroups with food groups.

Serogroups	Raw meat		Raw poultry		RTE food		Raw seafood	
	*n* (%)	*p*	*n* (%)	*p*	*n* (%)	*p*	*n* (%)	*p*
IIa	24 (57.4)		20 (44.44)		10 (32.26)		9 (45)	
IIb	3 (7.14)		10 (22.22)		15 (48.38)	<0.05	9 (45)	<0.05
IIc	14 (31.11)	<0.05	14 (33.33)	<0.05	2 (6.45)		1 (2.22)	
IVb	1 (2.38)		1 (2.22)		4 (12.49)		1 (2.22)	

*n*, number of isolates. %, the percentage of one serogroup isolates in all isolates from the same food group. *p* < 0.05 was regarded as statistically significance.

### Correlations Between CCs and Food Groups

**Figure 3:**
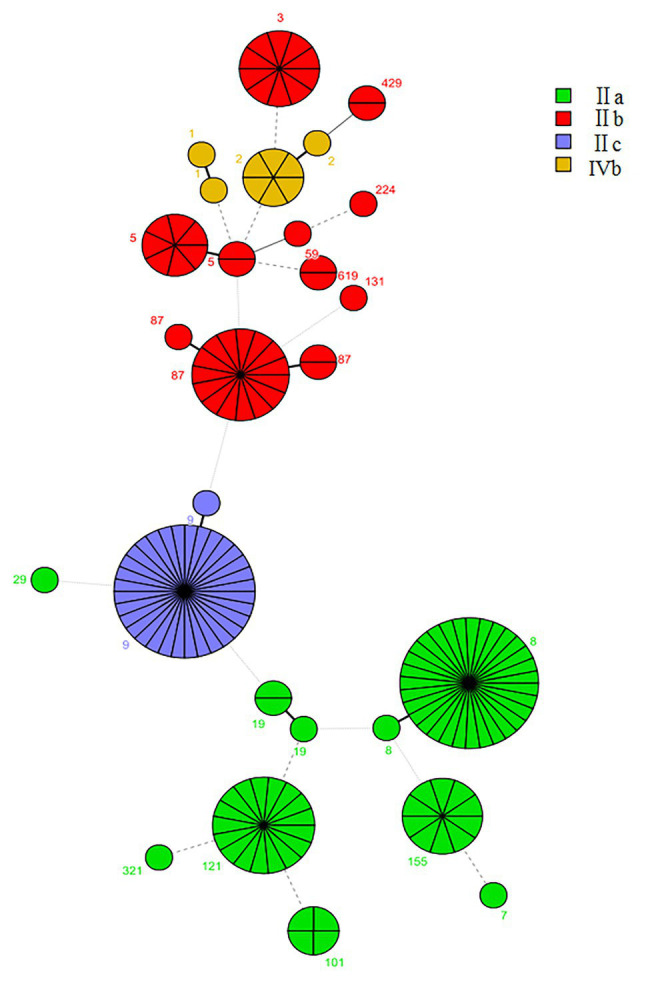
The minimum spanning tree of sequence types (STs) of 155 foodborne *L. monocytogenes* isolates from Shanghai, China. Each circle represents one ST, shown by clonal complex (CC). The circle size is proportional to the number of isolates and the color within the cycles represents the serotypes of the isolates. Links between circles are represented according to the number of allelic differences between STs.

Multilocus sequence typing detected 20 CCs and 27 STs in 155 *L. monocytogenes* isolates identified in this study, including two new STs (ST2105 and ST2106; Figure 3). The most common CCs were CC9 (including ST9 and the new ST2105) at 21.2% (33/155), CC8 (including ST8 and ST120) at 21.2% (33/155), CC87 (including ST87, ST310, and ST2106) at 11.6%, and CC121 (ST121) at 10.3%, followed by CC5 (including ST5 and ST1324), CC3 (ST3), CC155 (ST155), and CC2 (including ST2 and ST145) at 21.9%. Other CCs included CC101 (ST101), CC1 (ST1, ST515), CC19 (ST19, ST378), CC59 (ST59), CC619 (ST619), CC429 (ST429), CC29 (ST29), CC299 (ST299), CC224 (ST224), CC115 (ST115), CC321 (ST321), and CC7 (ST7; Figure 3). Each CC corresponded to a specific serogroup: IIa was the predominant serogroup of CC8, CC19, CC155, CC321, CC121, 101, and CC7. Serogroup IIb was the predominant serogroup of CC87, CC3, CC429, CC224, CC5, CC59, CC619, and CC131. Serogroup IIc had CC9. Serogroup 4b was the predominant serogroup of CC1 and CC2. Summarily, CC9, CC8, and CC87 were the predominant CCs in foodborne *L. monocytogenes* in Shanghai, China, and each CC corresponded to a unique serogroup.

The predominant CC distribution among different food groups was analyzed using a *t*-test (Table 2). The distribution of CC9 in raw meat and raw poultry significantly exceeded that of non-raw meat (*p* < 0.05). No significant differences in the distribution of CC8, CC121, and CC87 were observed in the four food groups.

**Table 2:** Pairwise association for *L. monocytogenes* CCs with food groups.

CCs	Raw meat		Raw poultry		RTE food		Raw seafood	
	*n* (%)	*p*	*n* (%)	*p*	*n* (%)	*p*	*n* (%)	*p*
CC9	14 (33.3)	<0.05	13 (28.8)	<0.05	2 (6.45)		1 (5)	
CC8	8 (19.05)		15 (33.33)	<0.05	3 (9.67)		2 (10)	
CC87	1 (2.38)		5 (11.11)		3 (9.67)		7 (35)	<0.05
CC121	9 (21.42)		3 (6.67)		2 (6.45)		2 (10)	

*n*, number of isolates. %, the percentage of one CC isolates in all isolates from the same food group. *p* < 0.05 was regarded as statistically significance.

### Comparison of Foodborne Isolates With Other Countries

**Figure 4:**
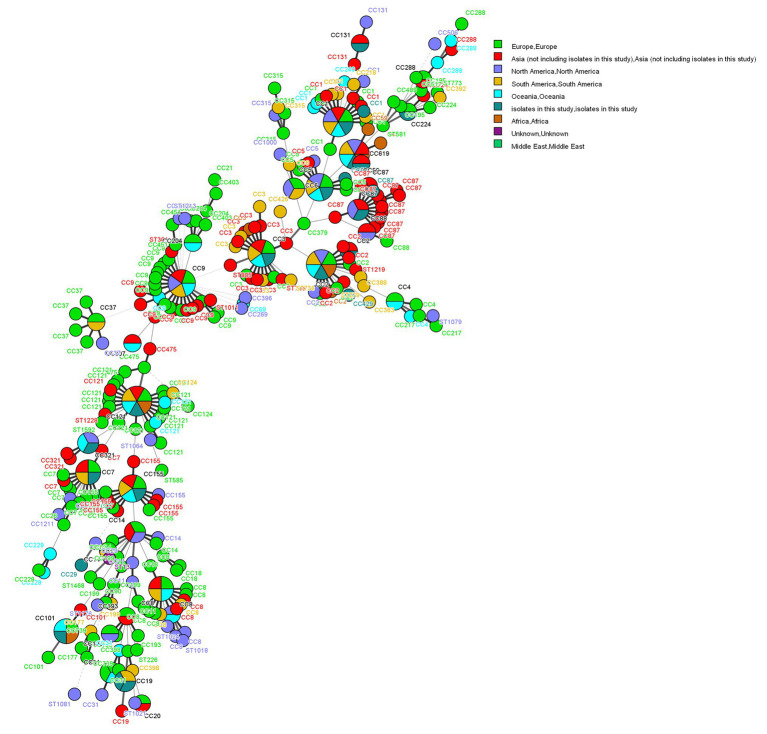
Phylogenetic trees constructed by UPGMA based on core genome multilocus sequence typing (cgMLST) loci. The corresponding data, including the name of the isolates (Key), MLST type (ST), CC, food groups, food category, year, and LIPI islands (LIPI-1–LIPI-4) are shown with the dendrogram to the right.

The 155 foodborne *L. monocytogenes* isolates were compared with isolates from Europe, Asia, North America, South America, Oceania, Africa, and the Middle East. Two isolates were from unknown areas (Figure 4), and 962 *L. monocytogenes* isolates from the *L. monocytogenes* MLST database^1^ as of 20 January 2020 were included in this analysis. The 1,117 foodborne isolates were divided into 66 CCs and 23 singletons. The most prevalent CCs in the database were CC3 (132), CC9 (129), CC121 (114), CC8 (93), CC1 (61), CC155 (63), CC2 (56), CC204 (39, not included in this study), and CC7 (34). All isolates obtained in this study, except CC429, were coclustered with foreign isolates. Eighteen CCs, except CC429 and CC619, detected in this study were also found in other countries (Figure 3).

The 1,117 *L. monocytogenes* isolates included nine CCs comprising more than 10 STs (Figure 4): CC9 (STs = 23), CC121 (STs = 17), CC3 (STs = 16), CC1 (STs = 14), CC8 (STs = 13), CC87 (STs = 13), CC155 (STs = 11), CC7 (STs = 10), and CC2 (STs = 10). The corresponding founders were ST9, ST121, ST3, ST1, ST8 ST87, ST155, ST7, and ST2.

### Presence of Genomic Islands in *Listeria monocytogenes*

**Figure 5:**
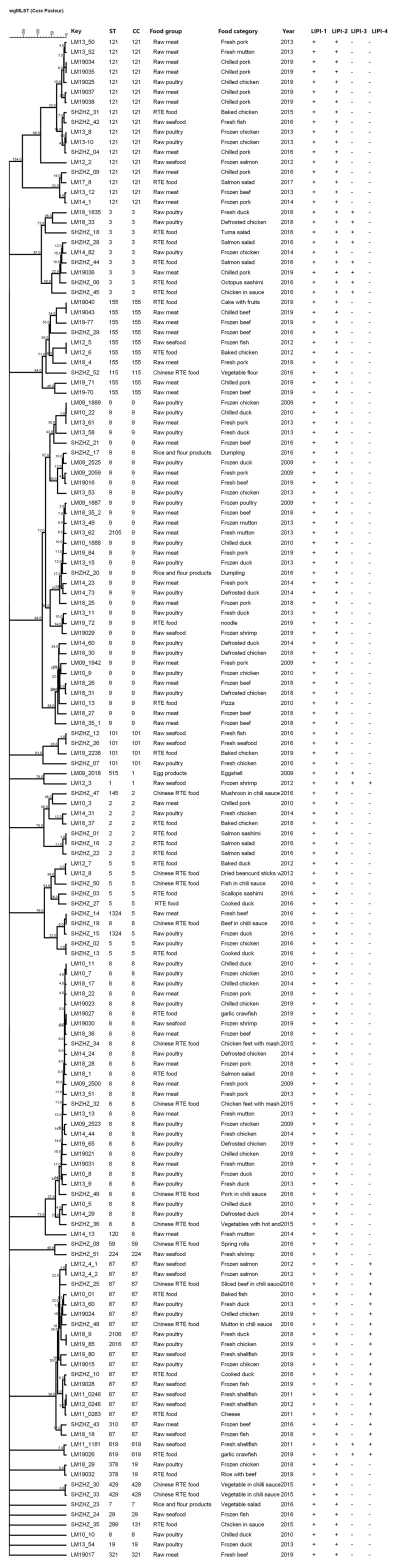
A minimum spanning tree was constructed based on CCs from this study and MLST database. The circle size is proportional to the number of isolates and the color within the cycles represents the source of the isolates.

In this study, all four known LIPIs were detected, and all *L. monocytogenes* isolates contained LIPI-1 and LIPI-2. However, only a few CCs contained LIPI-3 and LIPI-4 (Figure 5). LIPI-3 was detected in 10 CC3 and two CC1 isolates. Isolates with CC3 (IIb serogroup) were identified in five RTE foods, three samples of raw poultry, one sample of raw meat, and one Chinese RTE food. The two CC1 (4b) isolates were from raw seafood and eggshell. LIPI-4 was detected in 18 CC87 isolates, including ST87 and ST310. The 18 CC87 (IIb) isolates were from two RTE foods, three Chinese RTE foods, six raw poultry samples, and seven raw seafood samples. Also, LIPI-3 and LIPI-4 were detected in two CC619 (IIb) isolates from two raw seafood samples.

### cgMLST Typing

Twenty-one CTs were identified and found in different years and food groups (Figure 5). cgMLST analysis of *L. monocytogenes* isolates in CC9 showed 10–841 allelic differences (Figure 5). Three of them (LM09-1889, LM10-22, and LM13-61) showed 10 allelic differences. These isolates could be identified as the same clone (Ruppitsch et al., 2015) in different years and food products.

## Discussion

*Listeria monocytogenes* has emerged as an important foodborne pathogen. Nearly all reported listeriosis cases have been transmitted to humans *via* food (Scallan et al., 2011). In this study, seven food groups representing 22 food categories were analyzed for the molecular characterization of *L. monocytogenes* in Shanghai, China (Supplementary Table S1). The molecular characteristics of *L. monocytogenes* from meat products, RTE foods, and poultry products in China have been investigated (Zhang et al., 2019a). However, some foods have not been reported to be contaminated by *L. monocytogenes*. It was the first time that eggshell was reported to be contaminated by *L. monocytogenes*, which suggested that many food categories might be contaminated by *L. monocytogenes* due to cross-contamination, recontamination by products, environmental samples, and others. The contamination might occur in the market, supermarket, kitchen, or elsewhere.

From Figure 1, most *L. monocytogenes* isolates (79.3%, 123/155) from foods were detected from May to October (2009–2019). The seasonal variation was inconsistent with listeriosis occurrence in China (Chen et al., 2020c). Chen et al. analyzed the epidemiology of human listeriosis in China from 2008 to 2017, and reported that sporadic cases occurred from May to October (Chen et al., 2020c).

Identifying serogroups and CCs of *L. monocytogenes* is useful for initially identifying potential health risks that probably linked to food (Vongkamjan et al., 2017). Four serotypes (1/2a, 1/2b, 1/2c, and 4b) cause most cases of human listeriosis (Radoshevich and Cossart, 2017). Serogroup IIa and IIb isolates have been the cause of most human listeriosis cases, according to a national foodborne disease surveillance network (Li et al., 2019). The serogroup IIa was the most prevalent serogroup in most foods in China (Chen et al., 2019; Zhang et al., 2019a), also IIa strains have shown extensive distribution on various foods around the world (Korsak et al., 2012; Wu et al., 2015). Similarly, serogroup IIa and IIb were most prevalent, in this study. Furthermore, IIa isolates were distributed in various foods without significant differences. However, most serogroup IIb isolates in this study were isolated from RTE food (*p* < 0.05), which agreed with previous reports from China (Chen et al., 2020b).

For MLST analysis, 155 isolates belonging to 20 CCs, CC9, CC8, CC87, and CC121 (64.5%, 100/155), showed CC prevalence. The CC9 was most prevalent in raw meat and raw poultry (*p* < 0.05). Similarly, while CC8 in raw poultry was most prevalent than in RTE food and raw seafood, CC87 in raw seafood was most prevalent than the other three food groups (*p* < 0.05). However, CC121 was distributed in four food groups without significant differences. It has been reported that these CCs were associated with most food groups, such as meat and poultry products around the world (Henri et al., 2016; Sosnowski et al., 2019). Listeriosis was usually linked to consuming of contaminated food such as RTE meat foods, cheese, ice cream, and seafood (Todd and Notermans, 2011). However, listeriosis linked to consumption of raw meat and poultry has been rarely reported according to literature reports. Listeriosis might occur because of cross-contamination between raw meat and poultry. Raw seafood is usually the raw material used for producing RTE foods without heating, such as sushi and salad. Sporadic cases and outbreaks of listeriosis caused by seafood products have been reported (Riedo et al., 1994; Ericsson et al., 1997; Lyytikainen et al., 2006). Transfer of *L. monocytogenes* might therefore occur from contaminated raw seafood to RTE foods if the seafoods were produced under poor hygienic conditions (Mizan et al., 2015).

Most CCs in this study were linked to listeriosis cases in the world. CC87 was the most common CC in foodborne, clinical, and food-associated environments isolates in China (Chen et al., 2009; Pérez-Trallero et al., 2014; Wu et al., 2015). CC1 is the most prevalent genotype in Europe and America (Chenal-Francisque et al., 2011), and the CC1 prevalence in listeriosis cases has been reported (17-Chenal-Francisque et al., 2011; Huang et al., 2015; Kuch et al., 2018). A previous study conducted by us found that CC2, CC3, and CC5 were prevalent in foodborne and clinical isolates in Shanghai (unpublished). Li et al. (2018) has reported that these three CCs are prevalent in clinical isolates in China. The three CCs are strongly associated with listeriosis cases in other countries (Cartwright et al., 2013; Mentero et al., 2015). These data suggested that these CCs pose potential health risks to consumers.

Epidemiologically investigating of outbreaks of listeriosis has indicated that listeriosis cases have been linked to various food products, predominantly RTE foods with long shelf lives. However, in this study, most RTE foods and Chinese RTE foods had short shelf lives, usually less than 2 days of storage in a refrigerator. These foods were usually processed by small businesses. It appears that the application of food hygiene standards during processing of these foods was insufficiently stringent. Such products might cause most sporadic cases in China. Furthermore, these kinds of foods are consumed during a short period. Therefore, it is difficult to track the suspect food, which might cause listeriosis. These results probably explain why listeriosis outbreaks in China have not been reported until recently. It appears that RTE food and Chinese RTE food may be high-risk food items, and therefore, continuous surveillance of the molecular characteristics of *L. monocytogenes* in these two food groups is critical. Presumably, this study unprecedentedly detected *L. monocytogenes* from eggshells in China. However, Trudeau et al. (2020) confirmed that fecal microbial ecosystem of laying breeder hens could transfer to establish microbial on the surface of laid eggs, along with eggshell levels. Rivoal et al. (2013) has reported that raw egg products could be contaminated by *L. monocytogenes*, but no pasteurized egg products were contaminated by *L. monocytogenes* isolates. Therefore, strict adherence to standards of food hygiene is critical.

Our study showed that almost all CCs from foodborne *L. monocytogenes* isolates were detected in more than two countries. The exception was CC429 and CC619, which was reported only in China (Figure 4). *Listeria monocytogenes* is widely distributed globally, possibly due to its ability to adapt to different environments. *Listeria monocytogenes* CC87 was the most common CC in food products, human infections, and food-associated environments in China (Chen et al., 2009; Pérez-Trallero et al., 2014; Wu et al., 2015). However, CC87 has rarely been reported in other countries, excepting Spain (Wang et al., 2012; Rodríguez-Lázaro et al., 2015). Two outbreaks (both in Spain) caused by CC87 occurred in 2013 and 2014 (Pérez-Trallero et al., 2014). Therefore, CC87 might be the main CC causing listeriosis cases in China. Wang studied ST87 using WGS, and showed that all ST87 carried LIPI-4, a cluster of six genes encoding a cellobiose-family phosphotransferase system (PTS). This PTS has been identified as a hypervirulent factor implicated in maternal-neonatal and central nervous system infections (Maury et al., 2016; Wang et al., 2019). The presence of LIPI-4 might be linked to the prevalence of ST87 in sporadic outbreak cases. These results underscore the necessity of continuous surveillance of ST87 in food products and clinical cases in China. A novel type II RM system and novel plasmid were previously reported to be in ST87, apart from prophage P1 and other prophages in ST87 (Wang et al., 2019). Their significance is unknown, and requires further study.

Four *L. monocytogenes* pathogenicity islands have been identified (Cossart, 2011). LIPI-1 and LIPI-2 genes were detected in almost all *L. monocytogenes* isolates in this study. Many studies have shown that the hypervirulence exists in *L. monocytogenes* with different STs, and CC619 isolates carried LIPI-3 and LIPI-4. However, presumably, CC619 was detected in foodborne and clinical isolates only in China (Zhang et al., 2019b). However, CC619 was not prevalent either in foodborne isolates or in clinical isolates, although it carried all four LIPIs. In this study, although CC1 and CC3 carried LIPI-3, they were uncommon in clinical isolates, suggesting that the prevalence and pathogenicity of *L. monocytogenes* might also be related to CC serogroup. These results underscore the significance of molecular epidemiological surveillance of *L. monocytogenes* in foodborne products to assess the potential risk of *L. monocytogenes* and further address food safety issues in China.

In this study, we used cgMLST to perform cluster analysis, because cgMLST has higher discriminatory powers than MLST. Using cgMLST, 20 clusters were obtained (Figure 5), a finding that conformed to the presence of 20 CCs. However, allelic differences among isolates in the same cluster were significant. The allelic differences among CC87 isolates ranged from 0 to 360. The CC87 isolates from the same food groups in different years had no allelic differences, suggesting that the *L. monocytogenes* isolates could persist over years. These results indicate the existence of clonal transmission of CC87 in the environment. In this study, three CC9 isolates from different food groups over different years showed no allelic differences, further confirming the persistence and clonal transmission of *L. monocytogenes* isolates in the environment. These results indicate that WGS can be valuable in epidemiological surveillance of *L. monocytogenes* in China.

## Conclusion

A study of the molecular characteristics of 155 *L. monocytogenes* isolates from seven groups in 22 categories in Shanghai, China, was performed using WGS. Differences in the distribution of serogroups IIb, IIc, and IVb and CC9, CC8, CC87, and CC121 between food groups revealed that they had specific ecological niches. However, serogroup IIa was identified in different food groups, suggesting that this serogroup adapts readily to different environments. Hypervirulence was distributed in specific CCs, with LIPI-3 in CC3 and CC1, and LIPI-4 in CC87. CC619 carried LIPI-3 and LIPI-4. Analysis using cgMLST suggested that *L. monocytogenes* isolates could persist over years. These results underscore the significance of molecular epidemiological surveillance of *L. monocytogenes* in foodborne products, to assess the potential risk of *L. monocytogenes* and further address food safety issues in China.

## Data Availability Statement

The datasets presented in this study can be found in online repositories. The names of the repository/repositories and accession number(s) can be found in the article/Supplementary Material.

## Author Contributions

HZ and XZ designed the study, drafted and revised this manuscript, and analyzed data. CJ and JW performed whole-genome sequencing. BX and HL were involved in collection of isolates. QD revised this manuscript. All authors contributed to the article and approved the submitted version.

### Conflict of Interest

The authors declare that the research was conducted in the absence of any commercial or financial relationships that could be construed as a potential conflict of interest.

## Footnotes

^1^https://bigsdb.pasteur.fr/
