# Supplementary material for: 10-Year Molecular Surveillance of Listeria monocytogenes Using Whole-Genome Sequencing in Shanghai, China, 2009–2019
Source: Front Microbiol. 2020 Dec 15;11:551020. doi: 10.3389/fmicb.2020.551020 (PMC7769869; doi:10.3389/fmicb.2020.551020)
Supplement: Supplementary file 1 [file Table_1.docx]

Supplemental table. 1 The information of foods in this study

| **Key** | **Serogroup** | **ST** | **CC** | **Food group** | **Food category** | **Separating time** | **Accession number** |
| --- | --- | --- | --- | --- | --- | --- | --- |
| SHZHZ_01 | IVb | 2 | 2 | RTE food | Salmon sachimi | 2016-09 | JADKTQ000000000 |
| SHZHZ_06 | Ⅱb | 3 | 3 | RTE food | Octopus sashimi | 2016-08 | JADKTT000000000 |
| SHZHZ_44 | Ⅱb | 3 | 3 | RTE food | Salmon salad | 2016-06 | JADKXN000000000 |
| LM17_8 | Ⅱa | 121 | 121 | RTE food | Salmon salad | 2017-06 | JADKVU000000000 |
| LM18_1 | Ⅱa | 8 | 8 | RTE food | Salmon salad | 2018-05 | JADKVV000000000 |
| SHZHZ_16 | IVb | 2 | 2 | RTE food | Salmon salad | 2016-09 | JADKUC000000000 |
| SHZHZ_18 | Ⅱb | 3 | 3 | RTE food | Tuma salad | 2018-08 | JADKUE000000000 |
| SHZHZ_22 | IVb | 2 | 2 | RTE food | Salmon salad | 2016-07 | JADKUI000000000 |
| SHZHZ_23 | Ⅱa | 7 | 7 | Rice and flour products | Vegetable salad | 2016-08 | JADKUJ000000000 |
| SHZHZ_28 | Ⅱb | 3 | 3 | RTE food | Salmon salad | 2016-08 | JADKUO000000000 |
| SHZHZ_10 | Ⅱb | 87 | 87 | RTE food | Cooked duck | 2016-06 | JADKTX000000000 |
| SHZHZ_13 | Ⅱb | 5 | 5 | RTE food | Cooked duck | 2016-07 | JADKTZ000000000 |
| LM12_6 | Ⅱa | 155 | 155 | RTE food | Baked chicken | 2012-05 | JADKUY000000000 |
| LM12_7 | Ⅱb | 5 | 5 | RTE food | Baked duck | 2012-05 | JADKMV000000000 |
| LM10_13 | Ⅱc | 9 | 9 | RTE food | Pizza | 2010-06 | JADKXE000000000 |
| LM11_0283 | Ⅱb | 87 | 87 | RTE food | Cheese | 2011-01 | JADKUT000000000 |
| LM14_53 | Ⅱa | 8 | 8 | RTE food | Fresh tomato | 2014-04 | JADKVR000000000 |
| LM19_6 | Ⅱb | 3 | 3 | RTE food | Watermelon | 2019-03 | JADKML000000000 |
| LM19040 | Ⅱa | 155 | 155 | RTE food | Cake with fruits | 2019-07 | JADKTJ000000000 |
| LM19_32 | Ⅱa | 378 | 19 | RTE food | Rice with beef | 2019-06 | JADKMI000000000 |
| LM19_72 | Ⅱc | 9 | 9 | RTE food | Noodle | 2019-08 | JADKTN000000000 |
| SHZHZ_27 | Ⅱb | 5 | 5 | RTE food | Cooked duck | 2016-10 | JADKUN000000000 |
| LM18_37 | IVb | 2 | 2 | RTE food | Baked chicken | 2018-05 | JADKWJ000000000 |
| LM19_2238 | Ⅱa | 101 | 101 | RTE food | Baked chicken | 2019-05 | JADKMD000000000 |
| SHZHZ_31 | Ⅱa | 121 | 121 | RTE food | Baked chicken | 2015-05 | JADKUR000000000 |
| SHZHZ_35 | Ⅱb | 299 | 131 | RTE food | Chicken in sauce | 2015-05 | JADKXJ000000000 |
| SHZHZ_45 | Ⅱb | 3 | 3 | RTE food | Chicken in sauce | 2016-06 | JADKXO000000000 |
| LM10_01 | Ⅱb | 87 | 87 | RTE food | Chicken in sauce | 2010-03 | JADKWX000000000 |
| LM19_26 | Ⅱb | 619 | 619 | RTE food | garlic crawfish | 2019-06 | JADKSZ000000000 |
| LM19_27 | Ⅱa | 8 | 8 | RTE food | garlic crawfish | 2019-06 | JADKTA000000000 |
| SHZHZ_02 | Ⅱb | 5 | 5 | RTE food | Cooked duck | 2016-07 | JADKTR000000000 |
| SHZHZ_07 | Ⅱa | 101 | 101 | Raw poultry | Fresh chicken | 2016-09 | JADKTU000000000 |
| LM13-9 | Ⅱa | 8 | 8 | Raw poultry | Fresh duck | 2013-02 | JADKVA000000000 |
| LM13-11 | Ⅱc | 9 | 9 | Raw poultry | Fresh duck | 2013-02 | JADKVB000000000 |
| LM13-58 | Ⅱc | 9 | 9 | Raw poultry | Fresh duck | 2013-07 | JADKVI000000000 |
| LM13-60 | Ⅱb | 87 | 87 | Raw poultry | Fresh duck | 2013-07 | JADKVJ000000000 |
| LM14_31 | IVb | 2 | 2 | Raw poultry | Fresh chicken | 2014-04 | JADKVP000000000 |
| LM14_44 | Ⅱa | 8 | 8 | Raw poultry | Fresh chicken | 2014-05 | JADKVQ000000000 |
| LM18_9 | Ⅱb | 2106 | 87 | Raw poultry | Fresh duck | 2018-06 | JADKVW000000000 |
| LM18_1835 | Ⅱb | 3 | 3 | Raw poultry | Fresh duck | 2018-05 | JADKWK000000000 |
| LM19_85 | Ⅱb | 2016 | 87 | Raw poultry | Fresh chicken | 2019-09 | JADKMF000000000 |
| SHZHZ_15 | Ⅱb | 1324 | 5 | Raw poultry | Fresh duck | 2016-09 | JADKUB000000000 |
| LM09_1887 | Ⅱc | 9 | 9 | Raw poultry | Frozen poultry | 2009-08 | JADKWO000000000 |
| LM09_1889 | Ⅱc | 9 | 9 | Raw poultry | Frozen chicken | 2009-08 | JADKWP000000000 |
| LM09_2523 | Ⅱa | 8 | 8 | Raw poultry | Frozen chicken | 2009-10 | JADKWV000000000 |
| LM09_2525 | Ⅱc | 9 | 9 | Raw poultry | Frozen Duck | 2009-10 | JADKWW000000000 |
| LM10_7 | Ⅱa | 8 | 8 | Raw poultry | Frozen chicken | 2010-05 | JADKXA000000000 |
| LM10_8 | Ⅱa | 8 | 8 | Raw poultry | Frozen Duck | 2010-05 | JADKXB000000000 |
| LM10_9 | Ⅱc | 9 | 9 | Raw poultry | Frozen chicken | 2010-05 | JADKXC000000000 |
| LM13-8 | Ⅱa | 121 | 121 | Raw poultry | Frozen chicken | 2013-01 | JADKMT000000000 |
| LM13-10 | Ⅱa | 121 | 121 | Raw poultry | Frozen chicken | 2013-02 | JADKMS000000000 |
| LM13-15 | Ⅱc | 9 | 9 | Raw poultry | Frozen duck | 2013-03 | JADKVD000000000 |
| LM13-53 | Ⅱc | 9 | 9 | Raw poultry | Frozen chicken | 2013-06 | JADKVH000000000 |
| LM14_82 | Ⅱb | 3 | 3 | Raw poultry | Frozen chicken | 2014-08 | JADKMN000000000 |
| LM19_15 | Ⅱb | 87 | 87 | Raw poultry | Frozen chicken | 2019-04 | JADKMK000000000 |
| LM18_29 | Ⅱa | 378 | 19 | Raw poultry | Frozen chicken | 2018-07 | JADKMM000000000 |
| LM10_5 | Ⅱa | 8 | 8 | Raw poultry | Chilled duck | 2010-05 | JADKWZ000000000 |
| LM10_11 | Ⅱa | 8 | 8 | Raw poultry | Chilled duck | 2010-05 | JADKXD000000000 |
| LM10_1888 | Ⅱc | 9 | 9 | Raw poultry | Chilled duck | 2010-05 | JADKXG000000000 |
| LM10_22 | Ⅱc | 9 | 9 | Raw poultry | Chilled duck | 2009-06 | JADKXF000000000 |
| LM19_21 | Ⅱa | 8 | 8 | Raw poultry | Chilled chicken | 2019-06 | JADKWN000000000 |
| LM19023 | Ⅱa | 8 | 8 | Raw poultry | Chilled chicken | 2019-06 | JADKSW000000000 |
| LM19_24 | Ⅱb | 87 | 87 | Raw poultry | Chilled chicken | 2019-06 | JADKSX000000000 |
| LM19025 | Ⅱa | 121 | 121 | Raw poultry | Chilled chicken | 2013-06 | JADKSY000000000 |
| LM18_17 | Ⅱa | 8 | 8 | Raw poultry | Chilled chicken | 2018-08 | JADKVX000000000 |
| LM14_24 | Ⅱa | 8 | 8 | Raw poultry | Defrosted chicken | 2014-04 | JADKVN000000000 |
| LM14_29 | Ⅱa | 8 | 8 | Raw poultry | Defrosted duck | 2014-01 | JADKVO000000000 |
| LM14_60 | Ⅱc | 9 | 9 | Raw poultry | Defrosted chicken | 2014-02 | JADKVS000000000 |
| LM14_73 | Ⅱc | 9 | 9 | Raw poultry | Defrosted duck | 2014-06 | JADKVT000000000 |
| LM18_30 | Ⅱc | 9 | 9 | Raw poultry | Defrosted chicken | 2018-07 | JADKWE000000000 |
| LM18_31 | Ⅱc | 9 | 9 | Raw poultry | Defrosted chicken | 2018-07 | JADKWF000000000 |
| LM18_33 | Ⅱb | 3 | 3 | Raw poultry | Defrosted chicken | 2018-07 | JADKWG000000000 |
| LM19_65 | Ⅱa | 8 | 8 | Raw poultry | Defrosted chicken | 2019-08 | JADKTL000000000 |
| SHZHZ_04 | Ⅱa | 121 | 121 | Raw meat | Chilled pork | 2016-08 | JADKTS000000000 |
| SHZHZ_14 | Ⅱb | 1324 | 5 | Raw meat | Fresh beef | 2016-09 | JADKUA000000000 |
| LM09_1942 | Ⅱc | 9 | 9 | Raw meat | Fresh pork | 2009-08 | JADKWQ000000000 |
| LM09_2059 | Ⅱc | 9 | 9 | Raw meat | Fresh pork | 2009-09 | JADKWS000000000 |
| LM13-13 | Ⅱa | 8 | 8 | Raw meat | Fresh mutton | 2013-02 | JADKVC000000000 |
| LM13-50 | Ⅱa | 121 | 121 | Raw meat | Fresh pork | 2013-06 | JADKVF000000000 |
| LM13-51 | Ⅱa | 8 | 8 | Raw meat | Fresh beef | 2013-06 | JADKVG000000000 |
| LM13-52 | Ⅱa | 121 | 121 | Raw meat | Fresh mutton | 2013-06 | JADKMQ000000000 |
| LM13-61 | Ⅱc | 9 | 9 | Raw poultry | Fresh pork | 2013-07 | JADKVK000000000 |
| LM14_13 | Ⅱa | 120 | 8 | Raw meat | Fresh mutton | 2014-03 | JADKVM000000000 |
| LM14_23 | Ⅱc | 9 | 9 | Raw meat | Fresh pork | 2014-04 | JADKMO000000000 |
| LM19_16 | Ⅱc | 9 | 9 | Raw meat | Frozen beef | 2019-04 | JADKWM000000000 |
| LM19_17 | Ⅱa | 321 | 321 | Raw meat | Frozen beef | 2019-05 | JADKMJ000000000 |
| LM19_84 | Ⅱc | 9 | 9 | Raw meat | Fresh pork | 2019-09 | JADKTP000000000 |
| LM19_4 | Ⅱa | 155 | 155 | Raw meat | Fresh pork | 2019-03 | JADKWL000000000 |
| LM19_31 | Ⅱa | 8 | 8 | Raw meat | Fresh mutton | 2019-06 | JADKTE000000000 |
| SHZHZ_21 | Ⅱc | 9 | 9 | Raw meat | Frozen beef | 2016-08 | JADKUH000000000 |
| SHZHZ_29 | Ⅱa | 155 | 155 | Raw meat | Frozen beef | 2015-05 | JADKUP000000000 |
| SHZHZ_43 | Ⅱb | 310 | 87 | Raw meat | Frozen beef | 2016-04 | JADKXM000000000 |
| LM13-12 | Ⅱa | 121 | 121 | Raw meat | Frozen beef | 2013-02 | JADKMR000000000 |
| LM13-49 | Ⅱc | 9 | 9 | Raw meat | Frozen mutton | 2013-06 | JADKVE000000000 |
| LM09_2500 | Ⅱa | 8 | 8 | Raw meat | Fresh pork | 2009-10 | JADKWU000000000 |
| LM13-62 | Ⅱc | 2105 | 5 | Raw meat | Frozen mutton | 2013-07 | JADKVL000000000 |
| LM14_1 | Ⅱa | 121 | 121 | Raw meat | Frozen pork | 2014-01 | JADKMP000000000 |
| LM19_70 | Ⅱa | 155 | 155 | Raw meta | Frozen beef | 2019-08 | JADKTM000000000 |
| LM19_77 | Ⅱa | 155 | 155 | Raw meat | Frozen beef | 2019-09 | JADKMG000000000 |
| LM18_22 | Ⅱa | 8 | 8 | Raw meat | Frozen pork | 2018-07 | JADKVZ000000000 |
| LM18_25 | Ⅱc | 9 | 9 | Raw meat | Frozen pork | 2018-07 | JADKWA000000000 |
| LM18_26 | Ⅱc | 9 | 9 | Raw meat | Frozen beef | 2018-07 | JADKWB000000000 |
| LM18_27 | Ⅱc | 9 | 9 | Raw meat | Frozen beef | 2018-07 | JADKWC000000000 |
| LM18_28 | Ⅱa | 8 | 8 | Raw meat | Frozen pork | 2018-07 | JADKWD000000000 |
| LM18_35_1 | Ⅱc | 9 | 9 | Raw meat | Frozen beef | 2018-05 | JADKWH000000000 |
| LM18_35_2 | Ⅱc | 9 | 9 | Raw meat | Frozen beef | 2018-05 | JADKXY000000000 |
| LM18_36 | Ⅱa | 8 | 8 | Raw meat | Frozen beef | 2018-05 | JADKWI000000000 |
| LM19_71 | Ⅱa | 155 | 155 | Raw meat | Chilled pork | 2016-08 | JADKME000000000 |
| LM19034 | Ⅱa | 121 | 121 | Raw meat | Chilled pork | 2019-06 | JADKTF000000000 |
| LM19035 | Ⅱa | 121 | 121 | Raw meat | Chilled pork | 2019-06 | JADKMH000000000 |
| LM19036 | Ⅱb | 3 | 3 | Raw meat | Chilled pork | 2019-06 | JADKTG000000000 |
| LM19037 | Ⅱa | 121 | 121 | Raw meat | Chilled pork | 2019-06 | JADKTH000000000 |
| LM19_38 | Ⅱa | 121 | 121 | Raw meat | Chilled pork | 2019-06 | JADKTI000000000 |
| LM19043 | Ⅱa | 155 | 155 | Raw meat | Chilled beef | 2019-07 | JADKTK000000000 |
| LM10_3 | IVb | 2 | 2 | Raw meat | Chilled pork | 2010-03 | JADKWY000000000 |
| SHZHZ_19 | Ⅱa | 8 | 8 | Chinese RTE food | Beef in chili sauce | 2016-05 | JADKUF000000000 |
| SHZHZ_25 | Ⅱb | 87 | 87 | Chinese RTE food | Sliced beef in chili sauce | 2016-05 | JADKUL000000000 |
| SHZHZ_30 | Ⅱb | 429 | 429 | Chinese RTE food | Vegetable salad | 2015-05 | JADKUQ000000000 |
| SHZHZ_32 | Ⅱa | 8 | 8 | Chinese RTE food | Chicken feet with mashed garlic | 2015-07 | JADKXV000000000 |
| SHZHZ_33 | Ⅱb | 429 | 429 | Chinese RTE food | Vegetable salad | 2015-05 | JADKXH000000000 |
| SHZHZ_34 | Ⅱa | 8 | 8 | Chinese RTE food | Chicken feet with mashed garlic | 2015-05 | JADKXI000000000 |
| SHZHZ_36 | Ⅱa | 8 | 8 | Chinese RTE food | vegetables with hot and sour dishes | 2015-04 | JADKXK000000000 |
| SHZHZ_47 | 4b | 145 | 2 | Chinese RTE food | Mushroom in chili sauce | 2016-08 | JADKXP000000000 |
| SHZHZ_48 | Ⅱb | 87 | 87 | Chinese RTE food | Mutton in chili sauce | 2016-08 | JADKXQ000000000 |
| SHZHZ_49 | Ⅱa | 8 | 8 | Chinese RTE food | Pork in chili sauce | 2016-09 | JADKXR000000000 |
| SHZHZ_50 | Ⅱb | 5 | 5 | Chinese RTE food | Fish in chili sauce | 2016-10 | JADKXS000000000 |
| LM12_8 | Ⅱb | 5 | 5 | Chinese RTE food | Dried beancurd sticks with sauce | 2012-05 | JADKMU000000000 |
| SHZHZ_52 | Ⅱa | 115 | 115 | Chinese RTE food | Vegetable flour | 2016-10 | JADKXU000000000 |
| SHZHZ_09 | Ⅱa | 121 | 121 | Raw meat | Chilled pork | 2016-07 | JADKTW000000000 |
| SHZHZ_12 | Ⅱa | 101 | 101 | Raw seafood | Fresh fish | 2016-10 | JADKTY000000000 |
| LM09_2173 | Ⅱa | 101 | 101 | Raw seafood | Fresh clam | 2016-09 | JADKWT000000000 |
| SHZHZ_26 | Ⅱa | 101 | 101 | Raw seafood | Fresh seafood | 2016-10 | JADKUM000000000 |
| SHZHZ_42 | Ⅱa | 121 | 121 | Raw poultry | Frozen chicken | 2016-03 | JADKXL000000000 |
| SHZHZ_51 | Ⅱb | 224 | 224 | Raw seafood | Fresh shrimp | 2016-10 | JADKXT000000000 |
| LM11_0246 | Ⅱb | 87 | 87 | Raw seafood | Fresh shellfish | 2011-01 | JADKUS000000000 |
| LM11_1181 | Ⅱb | 619 | 619 | Raw seafood | Fresh shellfish | 2011-01 | JADKUU000000000 |
| LM12_0246 | Ⅱb | 87 | 87 | Raw seafood | Fresh shellfish | 2012-05 | JADKUZ000000000 |
| LM19_80 | Ⅱb | 87 | 87 | Raw seafood | Fresh shellfish | 2019-09 | JADKTO000000000 |
| LM12_2 | Ⅱa | 121 | 121 | Raw seafood | Frozen salmon | 2012-07 | JADKMX000000000 |
| LM12_3 | IVb | 1 | 1 | Raw seafood | Frozen shrimp | 2012-07 | JADKMW000000000 |
| LM12_4_1 | Ⅱb | 87 | 87 | Raw seafood | Frozen salmon | 2012-07 | JADKUV000000000 |
| LM12_4_2 | Ⅱb | 87 | 87 | Raw seafood | Frozen shrimp | 2012-07 | JADKUW000000000 |
| LM12_5 | Ⅱa | 155 | 155 | Raw seafood | Frozen fish | 2012-07 | JADKUX000000000 |
| LM18_18 | Ⅱb | 87 | 87 | Raw seafood | Frozen fish | 2018-08 | JADKVY000000000 |
| LM19_28 | Ⅱb | 87 | 87 | Raw seafood | Frozen fish | 2019-06 | JADKTB000000000 |
| LM19_29 | Ⅱc | 9 | 9 | Raw seafood | Frozen shrimp | 2019-06 | JADKTC000000000 |
| LM19_30 | Ⅱa | 8 | 8 | Raw seafood | Frozen shrimp | 2019-06 | JADKTD000000000 |
| SHZHZ_24 | Ⅱa | 29 | 29 | Raw seafood | Frozen fish | 2016-06 | JADKUK000000000 |
| SHZHZ_17 | Ⅱc | 9 | 9 | Rice and flour products | Dumpling | 2016-08 | JADKUD000000000 |
| SHZHZ_20 | Ⅱc | 9 | 9 | Rice and flour products | Dumpling | 2016-08 | JADKUG000000000 |
| SHZHZ_08 | Ⅱb | 59 | 59 | Chinese RTE food | Spring shrimp | 2016-11 | JADKTV000000000 |
| LM09_2018 | IVb | 515 | 1 | Egg products | Eggshell | 2009-09 | JADKWR000000000 |
